# Elaboration Benefits Source Memory Encoding Through Centrality Change

**DOI:** 10.1038/s41598-019-39999-1

**Published:** 2019-03-06

**Authors:** Inge K. Amlien, Markus H. Sneve, Didac Vidal-Piñeiro, Kristine B. Walhovd, Anders M. Fjell

**Affiliations:** 1 Center for Lifespan Changes in Brain and Cognition, University of Oslo, Oslo, Norway; 2 Department of Radiology and Nuclear Medicine, Oslo University Hospital, Oslo, Norway

## Abstract

Variations in levels of processing affect memory encoding and subsequent retrieval performance, but it is unknown how processing depth affects communication patterns within the network of interconnected brain regions involved in episodic memory encoding. In 113 healthy adults scanned with functional MRI, we used graph theory to calculate centrality indices representing the brain regions’ relative importance in the memory network. We tested how communication patterns in 42 brain regions involved in episodic memory encoding changed as a function of processing depth, and how these changes were related to episodic memory ability. Centrality changes in right middle frontal gyrus, right inferior parietal lobule and left superior frontal gyrus were positively related to semantic elaboration during encoding. In the same regions, centrality during successful episodic memory encoding was related to performance on the episodic memory task, indicating that these centrality changes reflect processes that support memory encoding through deep elaborative processing. Similar analyses were performed for congruent trials, i.e. events that fit into existing knowledge structures, but no relationship between centrality changes and congruity were found. The results demonstrate that while elaboration and congruity have similar beneficial effects on source memory performance, the cortical signatures of these processes are probably not identical.

## Introduction

The levels of processing (LOP) framework posits that the durability of a memory trace is a function of how deeply the memory has been encoded^1^. In general, we remember better when we learn using deep semantic processing than when we attend to shallower features, such as item color or shape. Behavioral evidence for these memory effects were first described decades ago^1–4^, and functional magnetic resonance imaging (fMRI) studies have provided support for the framework by shedding light on the neural components involved in the relationship between processing depth during encoding and successful memory formation^5,6^.

Episodic memory formation is dependent on the hippocampus and medial temporal lobe (MTL) structures^7,8^, while memory storage also depends on representations that are distributed across cortical networks, in a process of modality-specific cross-cortical storage^9^. Influential contemporary models of episodic memory function propose that events initially are encoded in parallel in both hippocampal and cortical networks of brain regions exterior to the MTL, including prefrontal cortex, bilateral fusiform, premotor, and posterior parietal cortex (see Kim^10^ and Paller^9^). Successful episodic memory encoding is thus dependent on the interactions between multiple spatially distributed cortical regions^11–13^.

Deep processing is associated with brain regions that are located in bilateral prefrontal cortex and hippocampus, while shallow processing is typically found to be supported by similar, but less spatially extensive regions than deep processing^14–18^. Neuroimaging has shown that the memory enhancing effect of processing depth may in part stem from increased functional connectivity between hippocampus and the discrete neocortical regions discussed above, possibly reflecting relational binding through hippocampal-cortical synchronization^6^. However, long-range integration and communication between neocortical regions outside the MTL could also be affected by different levels of processing. How the larger network of cortical regions involved in episodic memory encoding interacts during this process of initial binding of memory representations into coherent episodic memories is not yet well described.

Graph theory is a mathematical set of concepts and tools for network analyses that is well suited to describe and classify the roles different brain regions have in brain networks, and also how these roles may change in response to different situational demands^19^. A core graph theoretical concept concerns node centrality, i.e., the relative importance of a node and its capacity to influence other parts of the network^20^. Different measures of centrality can be applied to detect nodes that are highly connected and act like hubs, or nodes that are intermediate and rather affect the organization of the network^19,21^. Network characteristics during memory operations is modulated by memory state, for example whether an item can be retrieved or not, and the vividness of the retrieved memory. Successful retrieval has been linked to increased centrality in the left hippocampus^22^, and increased centrality and communication efficiency has been demonstrated during recollection of vivid vs. dim visual scenes^23^. Similarly, Schedlbauer *et al*.^24^ found that successful memory retrieval was associated with increased node centrality within the MTL, frontal and parietal lobes, and the visual cortex. Notably, this study also demonstrated that the increased centrality during successful retrieval was related to individual differences in retrieval accuracy.

Current research has focused on retrieval. However, the levels of processing framework include factors that probably act upon encoding more than retrieval, and processing depth during encoding may affect the strength of the memory trace and consequently subsequent memory success^1^. The beneficial effect processing depth has on episodic memory may be reflected through tighter integration and interaction between neocortical brain regions, and changes in the organization of the network of encoding-relevant regions during the initial binding of cortical memory representations. To examine levels of processing effects we thus have to focus on processes that occur during episodic memory encoding, rather than retrieval alone.

Elaboration and congruity are two related features of memory encoding that may have independent effects on memory strength^4,25^. While elaboration traditionally refers to how strongly a mental representation has been enriched during encoding by semantic evaluations^25^, congruity is a dimension of the LOP framework that refers to how well target words or items fit into existing knowledge structures or schemas^26^. Both elaboration and congruity are related to subsequent memory performance and can be operationalized as attributes of the encoding situation that can have different levels^2,5,25,27–29^.

Using a dataset that contains implicit information about levels of both congruity and elaboration, we examine systematic variation in levels of processing during encoding, and relate these processes to memory performance and measures of centrality (as outlined by Palombo and Madan^30^). We take advantage of the ratings across the elaboration- and congruity dimensions that the participants collectively and implicitly performed during a source memory encoding task. During encoding, the participants were shown 100 drawings of objects, and rated whether it was possible to eat or lift the items. We code the events where the participants agreed on the answer as low elaboration, e.g. “can you eat an apple?”, and we code events where the participants did not agree as high elaboration events, e.g. “can you lift a sofa?” We base congruity on the participants’ idiosyncratic responses, such that if a participant answered yes to the question “can you eat an apple?” that event would be coded as congruent, and vice versa.

We hypothesized that encoding of events that received deep processing (high elaboration or congruent trials) would be associated with increased centrality in nodes of the brain network that are predictive of successful episodic memory encoding.

To test this hypothesis, we employ a graph-theoretical approach^19,21,24^, and link fMRI blood oxygen level dependent (BOLD) activity and functional connectivity patterns to elaboration and congruity during source memory encoding. We generated seed regions from fMRI encoding activity that predicted subsequent source memory success. Using these seed points (including bilateral hippocampi) as nodes, we compared centrality during episodic memory encoding between different levels of elaboration and congruity. Next, for nodes that show increased centrality during high elaboration or congruity, we test if this functional reorganization of brain networks associated with different levels of processing can underlie parts of the beneficial effect processing depth has on episodic memory encoding. We ask whether increased centrality of these nodes during successful source memory encoding is associated with source memory performance, i.e. are the participants who are able to centralize these nodes during encoding also those who remember the most?

## Materials and Methods

### Sample

The study was approved by the Regional Ethical Committee of South Norway and all research was performed in accordance with relevant guidelines and regulations. All participants gave written informed consent. The participants reported no history of neurological or psychiatric disorders, chronic illness, premature birth, learning disabilities, or use of medicines known to affect nervous system functioning. They were further required to be right-handed, speak Norwegian fluently and have normal or corrected to normal hearing and vision. Participants were paid for their participation. 158 participants were scanned using fMRI while they were solving an item-action association task, and data from all these participants were used for the stimulus-classification (see methods/stimulus classification). 127 participants participated in an incidental memory test after a delay of 1.5 hours, and data from these participants were included the fMRI analyses. Four participants were excluded due to motion during the fMRI scan exceeding 1.5 mm (half the size of a voxel), or due to recalling less than 10% of the stimuli with full source memory. The sample thus consisted of 123 participants (female n = 89, age: range = 18.5–38.4 years, M = 27.8, SD = 5.15 [M = mean, SD = standard deviations]). We regarded less than ten trials in any of the categories used in the fMRI analyses (i.e. high/low elaboration/congruity, source memory correct/no source memory) to be insufficient for analysis, and ten participants who met these exclusion criteria were excluded from further analyses. A final sample of 113 participants (female n = 81 age: range = 18.5–38.4 years, M = 27.7, SD = 5.22) was thus used in the fMRI analyses.

### Procedure

**Figure 1:**
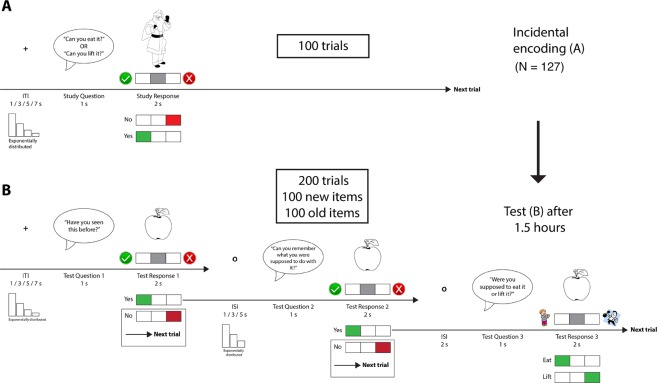
Schematic representation of the incidental encoding procedure (**A**), and the test procedure (**B**).

The memory task was optimized to allow for the investigation of individual differences in source memory performance, i.e. the ability to remember a previously encountered item together with information about the encoding context (an event). In line with recent theoretical accounts of source memory, our conceptualization of source memory considers all retained information about the encoding context as relevant, not only information about time and space e.g. Ranganath^31^. During encoding, participants went through 100 trials of a task in which they performed simple evaluations of everyday objects and items (Fig. 1A). A trial had the following structure: a black and white line drawing of an object was presented on the screen while a female voice asked either “Can you eat it?” or “Can you lift it?” Both questions were asked equally often and were pseudorandomly mixed across the different objects. Participants were instructed to produce yes/no-responses based on their subjective evaluations of object/task-contingencies, and that there were no correct responses to the task. Importantly, participants did not know that they were part of a memory experiment and would be tested on the evaluated material and remained ignorant about this until just before the test session. Responses were given by pressing buttons on the response grips according to on-screen instructions. The hand used to produce a “yes” response was counterbalanced between participants. After a response window of two seconds, the line drawing was replaced by a fixation cross which remained on screen during the interstimulus interval (ITI). ITI length varied randomly between 1–7 seconds with an exponential distribution over 4 discrete intervals (mean duration 2.98 s, SD = 2.49 s). The jittering of stimulus onsets facilitated later disentangling of fMRI data reflecting different encoding conditions^32,33^. Stimuli (10 visual degrees in diameter) were presented on a NNL 32″ LCD Monitor at a resolution of 1920 × 1080 pixels (NordicNeuroLab, Bergen, Norway), positioned 176 cm from the mirror attached to the coil. Participants responded using the ResponseGrip system (NordicNeuroLab, Bergen, Norway) and were shown a response feedback indicator on screen. Auditory stimuli were presented to the participants through scanner-compatible headphones (Siemens Medical Systems, Erlangen, Germany).

During test, 200 line drawings of objects were presented; 100 of these had been shown and evaluated during encoding while the remaining 100 objects were new (Fig. 1B). A test trial started with the presentation of an object (old or new, pseudorandomly picked) and the question “Have you seen this item before?” Participants were instructed to respond “Yes” if they remembered seeing the item during the encoding phase, and otherwise “No”. If the participant indicated that (s)he remembered seeing the object, a new question followed: “Can you remember what you were asked to do with the item?” A “Yes”-response to this question, indicating that the participant also remembered the action associated with the object during encoding, led to a final control question: “Were you asked to eat it or lift it?” Here, the participant indicated either “Eat” (“I evaluated whether I could eat the item during the encoding phase)” or “Lift” (“I evaluated if I could lift the item”). The test runs were also performed during fMRI in the same scanner. Note that the specific questions asked during scanning were simplified to fit within the temporal limits of the paradigm, but that all participants were instructed in detail before the test session that the questions pertained to the item-action evaluation performed at encoding. The task was originally described in Sneve *et al*.^13^.

### MRI data acquisition

A 3T Siemens Skyra system (Siemens Medical Systems, Erlangen, Germany) with a 24-channel Siemens head coil was used to acquire all MR images. The two encoding fMRI runs were acquired with the same parameters: 43 transversally oriented gapless slices were recorded using a BOLD-sensitive T2*-weighted echo planar image (EPI) sequence (repetition time [TR] = 2390 ms, echo time [TR] = 30 ms, flip angle = 90, voxel size = 3 × 3 × 3 mm, field of view [FOV] = 224 × 224 mm, interleaved acquisition (GRAPPA acceleration factor = 2, bottom ->up).

Three dummy volumes were collected at the start of each run, to avoid T1 saturation effects in the analyzed data. Each encoding run consisted of 131 volumes. A standard double-echo gradient-echo field map sequence was acquired for distortion correction of the EPI images. In addition, two sagittal T1-weighted MPRAGE volumes consisting of 176 sagittally oriented slices were obtained using a turbo field echo pulse sequence (TR = 2300 ms, TE = 2.98 ms, flip angle = 90°, voxel size = 1 × 1 × 1 mm, FOV = 256 × 256 mm). Several other MRI volumes were recorded during the session, not related to the current experiment, including sequences intended for and examined by a radiologist, to rule out and medically follow up incidental neuroradiological findings.

### Image analysis

FreeSurfer 5.3 was used for the cortical- and volumetric reconstruction of the T1-weighted structural data (http://freesurfer.net). The processing steps include motion correction and averaging^34^, removal of non-brain tissue^35^, automated Talairach transformation, and intensity correction^36^. Intensity and continuity information from the 3D volume are used in segmentation and deformation procedures to reconstruct a gray/white and gray/cerebrospinal fluid boundary throughout the brain^37–39^. Cortical surfaces then undergo inflation, registration to a spherical atlas, and identification of gyral and sulcal regions^40,41^. An experienced operator manually inspected individual surfaces and segmentations for accuracy, but no manual edits were deemed necessary. The FreeSurfer Functional Analysis Stream (FSFAST) v5.3 was used for preprocessing the functional image data from the encoding task. All functional images were first corrected for distortions caused by B0 inhomogenities in EPI scans (FSL PRELUDE/FUGUE; http://fsl.fmrib.ox.ac.uk/fsl), before the images were motion corrected (AFNI 3dvolreg; http://afni.nimh.nih.gov), slice timing corrected to the middle of a volume’s TR, intensity normalized, and registered to the same participants anatomical volume. The 4D functional data sets were then resampled to a common template (‘fsaverage’) using the surface-based inter-participant registrations created during the previous cortical reconstruction.

### Operationalization and analytical approach

The analytic approach we took was made in order to answer the two main hypotheses:

1. Encoding of events requiring deep processing (high elaboration or congruent trials) is associated with increased centrality in parts of the brain network involved in episodic memory encoding.
2. High centrality in levels of processing-hubs (i.e., brain regions that show increased centrality during deep processing, cf. hypothesis 1) is related to episodic memory performance.

**Figure 2:**
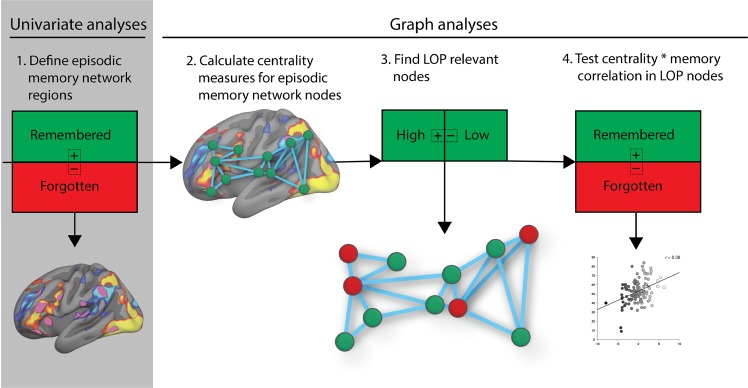
Schematic representation of the analytical approach. (1) We first defined regions that were involved in episodic memory encoding by contrasting events that were subsequently remembered with full source information (i.e. the participant remembered whether they answered the lift or eat question) with those that were not. 40 nodes, including both hippocampi brought to the graph analyses. (2) centrality measures were calculated for all nodes (closeness centrality, node degree, eigenvector centrality, betweenness centrality), (3) and we identified nodes that increased centrality during deep processing. (4) Finally, in the levels-of-processing-nodes identified in the previous step, we tested the correlation between A: centrality change between events that were subsequently remembered vs forgotten, and B: source memory performance. Plus/minus signs denotes contrasts. Node drawings are for illustration purposes only, and are not accurate representations of the node matrix.

A general overview of the analyses is presented in Fig. 2:

1. We identified regions (nodes) that are involved in episodic memory encoding by performing a whole-brain fMRI analysis, isolating positive and negative subsequent source memory effects. The resulting statistical maps were used as the basis for creating nodes that were inputs to the graph theoretical analyses. See below for details.
2. We calculated centrality measures for all nodes defined in (1) plus bilateral hippocampus, 42 nodes in total. Centrality was calculated for all conditions (high elaboration remembered, low elaboration remembered, high elaboration forgotten, low elaboration forgotten [and the same for congruity]).
3. We identified nodes that showed increased centrality during deep processing-trials.
4. Using the LOP nodes defined in the previous step, we tested the correlation between A: centrality change between events that were subsequently remembered vs forgotten, and B: source memory performance.
5. We permuted the analysis in the previous step, testing the likelihood of finding a similarly strong relationship between centrality and memory ability in any other constellations of nodes than the ones we found were relevant for LOP.

### Levels of Processing

In order to extract information about levels of processing from the data, we ranked the 100 events (item/question combinations) that were presented during encoding according to the total number of “yes” responses across all participants. Our conceptualization of elaboration involved that when an event was encountered for which the participants collectively agreed on the answer, less elaboration was required than for events where the participants where split in their decisions, i.e. where for example 50% answered “yes” and 50% “no”. High and low elaboration categories thus represent degree of agreement across subjects.

We envisioned that the events with low agreement across participants received a mix of yes and no responses because the item-action combinations were novel, conflicting, or evoked ambivalence. The increased time the participants spent on the high elaboration events before concluding with a push on the response button could thus reflect a qualitatively different form of semantic evaluation than for the low elaboration events, and possibly greater number of encoded features^2^. While the low elaboration events easily fit with previous experiences, either because the item-action combinations had been encountered previously, and the decision merely hinged on recollecting the episode, or the item-action combination was easily imaginable (i.e. can you lift an apple), the high elaboration events were the opposite. High elaboration events were assumed to demand a greater number of semantic iterations and possibly a greater number of encoded features, thus more effortful semantic processing before a decision could be reached, either because the item-action combinations were novel, or evoked cognitive conflicts. Examples of low elaboration events are the item “apple” combined with the eat-question, which all participants agreed was possible, and the item “Boat” combined with the eat-question, which all participants agreed was impossible. A high elaboration example is the item “sofa” combined with the lift-question, an event that received approximately 50/50 yes and no answers, reflecting that the participants probably had to perform some degree of semantic judgements on the item-question combination (for example evaluating whether they have lifted sofas in the past, or how heavy this specific sofa appears) before reaching a decision.

**Figure 3:**
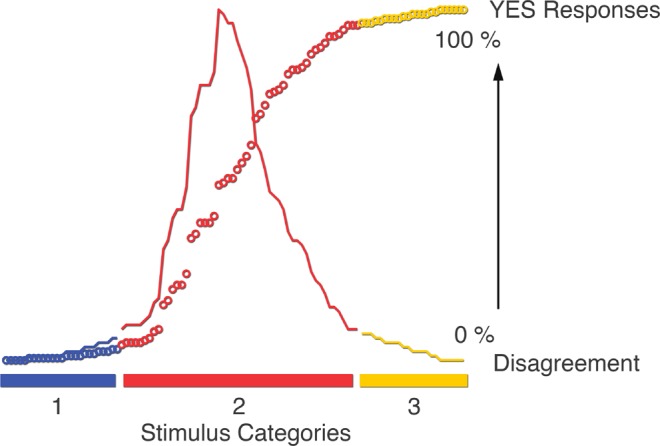
Stimulus classification categories. The stimulus items were ranked based on the sum of Yes-responses each item received during encoding (along the X-axis). The Y-axis represents percent Yes-responses per item (circles), and the amount of disagreement among the participants per item (solid line). Categories 1 and 3 are classified as low elaboration, category 2 as high elaboration.

The ranking and categorization of events is explained in Fig. 3. The events that received predominantly either “Yes” or “No” responses were categorized as low elaboration, and the events that the participants disagreed on were categorized as high elaboration. We performed the categorization by splitting the events into 4 quartiles based on total number of “yes” responses, and labelled the middle two quartiles that received mixed responses as high elaboration, and the top and bottom quartiles (mainly yes- and no responses) as low elaboration (Fig. 3).

Congruity was based solely on the participants’ idiosyncratic responses. Following Schulman^26^, we envisioned that item-action combinations that were congruent according to the individual participants mental representations, would be judged as possible more often than not, and be awarded with “yes” responses. When a positive response is made, the encoding question (lift/eat) and target (item) could form a more coherent and thus easier integrated unit. The congruity dimension was thus classified on the individual level, where the events the individual participant responded with “yes” were classified as high congruity events, and the events that received “no” responses were classified as low congruity events.

### Memory performance

A participant’s source memory score was calculated as the percentage of encoded events that were recognized with correct recollection of the associated encoding action. Thus, participants had to correctly recognize an item (correct “Yes” response to test question 1), state that they remembered the associated action (“Yes” response to test question 2), and explicitly pick the correct associated action (correct response to question 3) for that event to fall into the source memory category. The participant’s recognition score was the percentage of encoded items they correctly recognized (correct “Yes” response to test question 1), regardless of response to follow-up questions. As the number of events varied between individuals and categories, we performed analyses using ratio-scores (hit rate/number of events per condition) in the analyses of memory performance. Where applicable, we also tested d-prime scores in the recognition memory analyses, taking into account false alarm rates, and corrected source memory performance scores when possible (source memory performance - number of wrong action decisions, i.e. wrong answer to question 3). To correct for multiple tests, we used Bonferroni adjusted alpha levels, adjusted for four analyses (elaboration and congruity by recognition and source memory).

### fMRI analyses

For each encoding run, three first-level general linear models (GLM) were set up. First, we contrasted events subsequently remembered with full source memory with events without full source memory (i.e. recognition misses, recognition hits, no source memory, or wrong source memory). Two additional models were set up, with four main conditions/regressors (high elaboration remembered with full source memory, high elaboration without full source memory, low elaboration remembered with full source memory, low elaboration without full source memory, and similar for congruity. The regressors were modelled as events with onsets and durations corresponding to the encoding period (2 s), and convolved with a two-gamma canonical HRF. In addition to the task-regressors and their temporal derivatives, estimated motion correction parameters and a set of polynomials (up to second degree) were included in the GLM as nuisance regressors. The model and the data were high-pass filtered with a cutoff at 0.01 Hz, and temporal autocorrelations (AR(1)) in the residuals were corrected using a pre-whitening approach. Statistical significance was tested at each vertex on the cortical surface using GLMs and a weighted least squares approach^42^. For each individual, the contrasts of parameter estimates were calculated and brought to the group-level, where the following analyses were performed.

### Node matrix creation

We performed a whole-brain cortical GLM where we contrasted source memory > no source memory trials (i.e. recognition only or miss), regardless of elaboration or congruity status. These results provided a set of regions relevant for source memory encoding that were used for defining the nodes that were used in the graph analyses. Nodes were created by running the FDR corrected (*p* < 0.05) statistical map of the [source memory > no source memory] contrast through mri_surfcluster and saving clusters with an extent greater than 100 vertices. We algorithmically searched for local maximas in each cluster, restricted to minimum 25 mm separation between maximas, expanded the cluster down the gradients starting from the maxima following the cluster structure up to a maximum of 600 vertices, and saved the resulting ROIs. The specific values for minimum/maximum cluster size and degree of separation were chosen to keep nodes from overlapping and account for registration inaccuracies/smoothing. We included the set of nodes generated by this analysis, as well as the FreeSurfer-segmented left and right Hippocampus, in the following graph analyses. The reason we included the hippocampus in the analyses a priori was that the other nodes were derived from vertex-wise surface-based data, and FreeSurfer handles hippocampus in volume-space.

### Graph construction

The pre-processed functional data were analyzed for task-specific connectivity using the generalized psychophysiological interactions (PPI) toolbox^43^. First, observed BOLD data (first eigenvariate) for all nodes defined in the previous step were deconvolved into estimates of neural events^44^. Next, the task time courses from the first-level FSFAST design matrix, representing the stimulus categories (high and low elaboration, subsequently remembered, and similar for congruity), were separately multiplied by the deconvolved neural estimate, and convolved with a canonical HRF, creating the PPI terms. Finally, for each participant and condition, we calculated the Fisher-transformed temporal correlations (Pearson’s *r*) between all nodes’ PPI terms, creating a correlational PPI connectivity matrix of all pairwise combinations^45^. Due to a different number of trials going into each condition, that could also vary across participants (different number of yes/no responses), we thresholded all connectivity matrices to contain only the strongest edges. To allow comparisons between participants and conditions independent of absolute connectivity levels, we binarized the thresholded connectivity matrices. All centrality analyses were run on these undirected, thresholded, binarized connectivity matrices.

### Centrality measures

Centrality measures were calculated using the Brain Connectivity Toolbox (brain-connectivity-toolbox.net). We calculated four different but related measures of centrality: closeness centrality, degree, betweenness centrality and eigenvector centrality. The first three centrality measures were first proposed by Freeman^46^ and eigenvector centrality by Bonacich^47^. The application of the centrality measures for research on brain networks is further described in for example Van Den Heuvel *et al*.^48^.

#### Closeness Centrality (CC)

A node high in CC has on average shorter paths to other nodes in the network, than nodes low on CC. Both degree and closeness centrality are measures sensitive to global properties of the network and yield information about how easily information can spread from one node through the network. The CC of a network node can be calculated by the inverse of the sum of the shortest distance between the node and all other nodes in the graph:

$$CC(v)=\frac{1}{{\sum }_{i\ne v}{d}_{vi}}$$

All the analyzed measures of centrality (closeness, degree, betweenness, and eigenvector) are measures that describe aspects of a node’s importance in a network^49^. While degree and closeness are highly intercorrelated^50^, closeness reflects more global aspects of how a node relates to a network than the degree measure which simply counts number of connected edges. We chose to focus on CC in the main analyses, as this measure may correspond to more global aspects of network connectivity than node degree. However, as the different measures of centrality can be informative of different aspects of network organization, we include results from alternative measures of centrality as supplementary material (node degree, betweenness centrality [BC], and eigenvector centrality [EC])^48^.

### Graph centrality statistical analyses

To test whether any of the source memory encoding nodes increased centrality during deep processing, we conducted paired-samples t-tests on centrality for all 42 nodes. Comparisons were made between the high- and low-elaboration categories and between the high- and low-congruity categories that were subsequently recollected with source memory. To test if effects of levels of processing were simply effects of successful encoding, we also performed paired-samples t-tests on centrality for the difference memory contrast, between items that were remembered with full source memory and items that were not remembered with full source memory.

We performed analyses on binarized graphs that were thresholded using density based thresholding. Use of density-based thresholding allowed us to be able to match connection density across all participants. The most conservative threshold we employed was to keep 20% of the strongest edges, and this value was selected based on the criterion that at least 85% the nodes had at least one remaining edge, in at least 85% of the participants. In order to reduce the likelihood of type 1 errors due to spurious effects appearing at specific graph thresholds only, we performed all graph analyses on binarized graphs that were thresholded at 20, 22, 24, 26, 28 and 30% edge density, and only results that were significant (FDR-corrected at *p* < 0.05) across all six graph thresholds are reported and considered significant. This correction is quite conservative, and we can be fairly certain that any node that shows increased centrality in a condition is not due to chance alone.

To test the relationship between centrality and memory performance, we correlated source memory performance with average centrality change across the set of nodes that remained significant after the combined FDR correction and conjunction test across all thresholds. The analyses were performed on CC in the main analyses, with other graph centrality measures shown in supplementary material. To quantify the likelihood of obtaining similar results with any other constellation of brain regions, we compared the results with distributions of correlation coefficients derived from all possible constellations of the same number of randomly selected nodes (excluding the set of nodes nodes found in the previous analyses). The number of permutations in the exhaustive permutation tests were 73815 for CC (3 LOP nodes excluded), and 9139 for degree and EC (set of 4 nodes excluded). The 40 surface-based nodes are included in supplementary material on FreeSurfer annotation format.

## Results

### Stimulus classification

The 100 events presented during encoding were classified by ranking each events according to the accumulated number of “yes” responses across all participants (Fig. 3). For the low elaboration events, participants produced an average of 97.8% (SD = 1.6%) “yes” responses (Fig. 3, category 3) or 99.2% (SD = 0.7%) “no” responses (Fig. 3, category 1). Events classified as high elaboration i.e. combinations characterized by a high degree of disagreement, had on average 48.0% (SD = 32.1%) “yes” responses (Fig. 3, category 2). Congruity was classified on an individual basis, simply as the events where the participant answered “yes” (high congruity), or “no” (low congruity)^26^. There was a statistically significant difference in mean response time (RT) between high (mean = 1034 ms, SD = 131 ms) and low elaboration (mean = 848 ms, SD = 104 ms, *p* < 0.001), and between high (mean = 981 ms, SD = 125 ms) and low congruity (mean = 955 ms, SD = 124 ms, *p* < 0.001). The pattern of RTs reflects what would be expected from LOP theory, with increased RTs for high elaboration vs. low elaboration, and while previous studies have found small or no differences between high and low congruity conditions, the absolute differences we report were small^2^.

### Memory performance

Paired samples t-tests were performed on corrected source memory performance scores between high- and low elaboration, and high- and low congruity, separately. The analyses revealed that events processed with high elaboration were recollected more often with full source memory (mean = 64.09%, SD = 15.00%, t[122] = 24.09, *p* < 0.001) than events processed with low elaboration (mean = 35.52%, SD = 16.73%). This pattern held also for congruity, and a higher number of high congruity events were recollected with full source memory (mean = 56.93%, SD = 15.89%, t[122] = 11.68, *p* < 0.001), than low congruity events (mean = 42.61%, SD = 16.39%). The memory performance of the group reflected what we would expect according to LOP, with both elaboration and congruity positively related to source memory^2^. A similar pattern emerged when we analyzed differences in simple recognition, accounting for false positive rates. High elaboration was associated with better recognition (d′ = 2.05, SD = 0.48) than low elaboration (d′ = 1.71, SD = 0.56, t[122] = 14.05, *p* < 0.001), and the participants recognized the high congruity events more readily (d′ = 1.57, SD = 0.53) than the low congruity events (d′ = 1.30, SD = 0.53, t[122] = 12.82, *p* < 0.001)

### Centrality analyses

**Figure 4:**
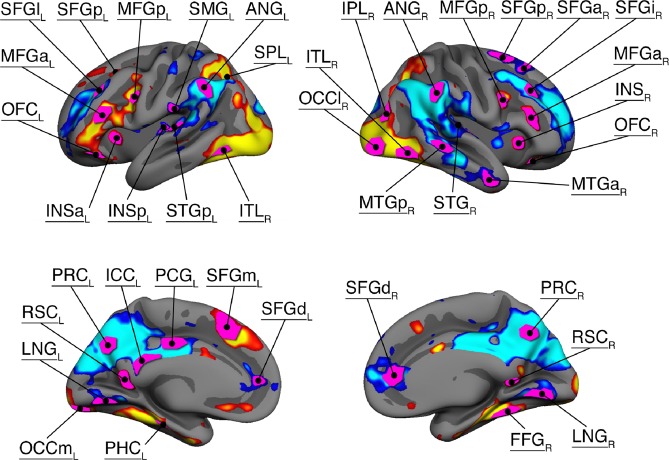
Areas showing significant subsequent source memory effects on BOLD activity, independent of elaboration and congruity. Nodes entered into the connectivity analyses shown in pink (Right and left Hippocampus not shown). The abbreviations for the nodes are as follows: SFG (Superior frontal gyrus), MFG (Middle frontal gyrus), SMG (Surpamarginal gyrus), ANG (Angular gyrus), SPL (Superior parietal lobe), ITL (inferior temporal lobe), MTG (Middle temporal gyrus), STG (Superior temporal gyrus), INS (Insula), OFC (Orbitofrontal cortex), IPL (Inferior parietal lobe), OFC (Orbitofrontal cortex), PRC (Precuneus), RSC (Retrosplenial cortex), ICC (Isthmucingulate cortex), PCG (Posterior cingulate cortex), PHC (Parahippocampal cortex), LNG (Lingual gyrus), FFG (Fusiform gyrus). The FreeSurfer annot-files describing the labels are available as Supplementary Material.

We defined a set of regions involved in source memory encoding that resulted in a network of 40 cortical nodes (Fig. 4). We also included the left and right Hippocampus, yielding a total of 42 nodes that were entered in the connectivity analyses. PPI terms were extracted from all nodes, for both high and low levels of elaboration and congruity, for the trials that were subsequently remembered with source memory, resulting in 42 × 42 correlation matrices per condition, per participant. We also extracted PPI terms for successful vs unsuccessful source memory encoding contrast, regardless of elaboration or congruity.

**Figure 5:**
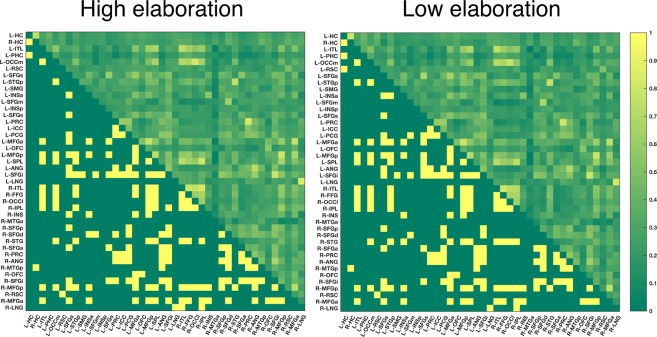
Node adjacency matrix showing the centrality structure during high and low elaboration, based on the group average. Raw connectivity weights between nodes above the diagonal, binarized edges at 20% edge density threshold below the diagonal.

To characterize how the brain’s nodal centrality characteristics are reorganized during different encoding conditions, we performed paired-samples t-tests on individual estimates of closeness centrality between conditions, across all nodes. We only report the nodes that were significant after FDR-correction at *p* < 0.05, at all six graph density thresholds (20%, 22%, 24%, 26%, 28%, 30%). An example graph matrix binarized at 20% threshold and the underlying raw connectivity values between nodes is shown in Fig. 5.

**Figure 6:**
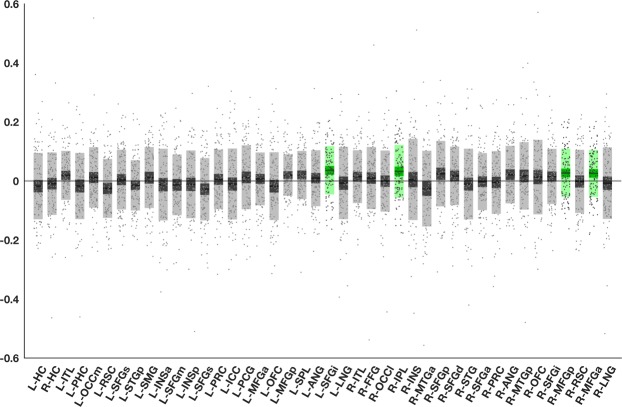
Nodes (X-axis) in green show significantly increased closeness centrality in the high-elaboration vs low elaboration condition. The values on the Y-axis represent closeness centrality change between conditions, averaged across all thresholds. Individual values are represented by the dots, horizontal lines represent group means, dark area = 95% confidence interval, light area = standard deviation.

**Figure 7:**
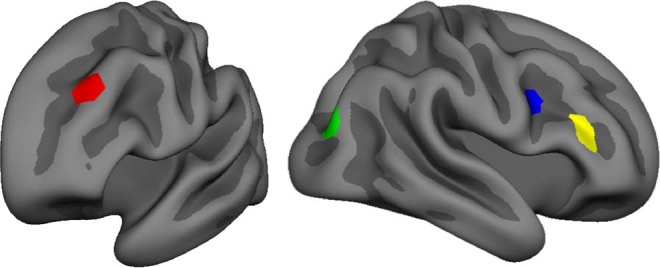
Nodes that show significantly increased CC in the high-elaboration vs low elaboration condition. Red = Left superior frontal gyrus - inferior; Green = Right inferior parietal lobule; Blue = Right middle frontal gyrus–posterior; Yellow = right middle frontal gyrus–anterior. Nodes are displayed on a semi-inflated FreeSurfer fsaverage template.

We found that CC was increased during the high elaboration condition compared to the low elaboration condition in three frontal nodes (left superior frontal gyrus-inferior node[L-SFGi], right middle frontal gyrus–anterior node [R-MFG-a] and the right middle frontal gyrus–posterior node [R-MFG-p]) and one posterior node (right inferior parietal lobule [R-IPL]). Plot of CC values for all nodes are shown in Fig. 6, and these four elaboration network nodes are display in Fig. 7. No nodes showed the opposite effects, i.e. higher CC during low elaboration compared to high elaboration. Three nodes (L-SFGi, RIPL and R-MFGp) also showed stability across three of the tested centrality measures (CC, degree, EC). No significant effects were found when comparing centrality during encoding of congruent vs incongruent events, or when testing subsequent source memory effects on centrality, regardless of levels of processing (i.e. comparing centrality during encoding of events that were subsequently remembered with source memory with events that were not remembered with source memory).

### Centrality and Source Memory

Elaboration is positively related to source memory performance, and the pattern of edge reorganization we detected as increased centrality during high elaboration could represent features of deep processing that gives rise to the beneficial effect on source memory encoding. Centrality differences in the elaboration network nodes during successful compared to unsuccessful source memory encoding could thus be indicative of the effectiveness of which participants use elaborative encoding strategies or processes when encoding memories. To test for the existence of such a relationship, we correlated mean centrality change in the elaboration network nodes during successful vs unsuccessful source memory encoding with individual source memory performance.

**Figure 8:**
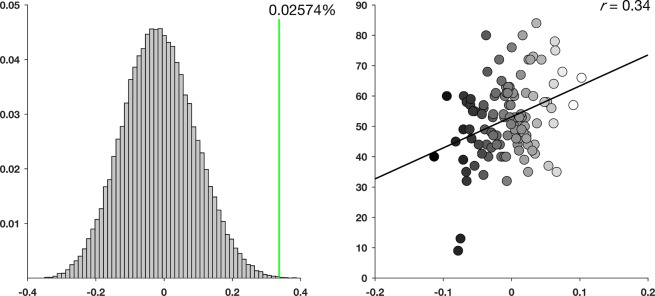
Left column; Null distribution of *r*-values (73815 permutations) between connectivity and source memory, using constellations of nodes not in the elaboration network. Correlation value from the right panel shown as green line. Right column; Mean centrality difference between subsequent source- and no subsequent source memory across elaboration network nodes of interest represented on the X-axis. Corrected source memory performance on the Y-axis.

There was a positive relationship between centrality during successful vs. unsuccessful source memory encoding and source memory performance (mean CC across the four elaboration network nodes: r = 0.34, *p* < 0.001, Fig. 8, right column). To test if this relationship was truly unique to the four elaboration network nodes or if a similar relationship could be identified also in other nodes, we performed the same tests in all 73815 possible unique permutations of subsets of four random nodes, excluding the previously identified four elaboration network nodes. We calculated centrality difference between source memory correct vs incorrect, across the six different thresholds as in the main analyses, averaged over the four randomly chosen nodes, and correlated the centrality values with source memory performance. This procedure was performed using every possible constellation of nodes, and the null-distribution of correlations between source memory performance and CC for all permutations was saved. The relevant correlation from the nodes in the elaboration network is plotted as the green line in Fig. 8, left column. Only 19/73815 unique permutations of nodes resulted in similar or higher correlation values, translating to 0.026% of the possible combinations of nodes. Comparable results were found for degree and EC (Fig. S5). A relationship with similar strength between source memory performance and increased centrality in the elaboration network nodes is thus not likely to be found in other subsets of nodes.

## Discussion

In this study, using a graph theoretical approach, we were able to identify a set of cortical regions involved in deep processing during associative encoding. The magnitude of centrality increase in these regions during successful encoding was positively related to individual source memory performance. The findings demonstrate that elaboration may benefit episodic memory encoding through centrality changes in a set of frontal and parietal brain regions. While several studies have shown how brain activity^5,17,51^ and connectivity^6^ is affected by changes in levels of processing, the present study attempts to unify these findings by using graph theoretical constructs to assess changes in brain network interaction and reorganization during episodic memory encoding with different levels of processing.

Successful episodic memory retrieval has been associated with changes in communication patterns of the brain, exemplified by increased centrality of distinct brain regions, possibly reflecting increased integrative processing^22,24^. While we were unable to detect equivalent centrality changes specific to successful source memory processes during encoding, a distinct set of nodes showed increased centrality during encoding of high elaboration vs low elaboration events.

Both the middle frontal gyrus and the posterior parietal cortex are involved in episodic memory encoding^10,52,53^ and retrieval^54^. Univariate BOLD activity in the posterior parietal cortex (R-IPL) is possibly related to search for task-related information in episodic memory during retrieval, and may be involved in binding features episodic memories to coherent wholes^55^. The IPL is also referred to as part of a network involved in various forms of scene processing and integration^56,57^, and related, as part of the posterior medial memory system thought to support the construction of situation models in support of memory^31^. It is likely that construction of scenes or situational models of events with high elaboration requires greater cognitive effort, or a more comprehensive memory search, than similar scene construction with low elaboration. The centrality increase in posterior parietal cortex we found in the present study during high elaboration may thus reflect increased network integration in networks involved in scene construction or the posterior medial memory system. As scene construction involves integrating episodic memory traces into coherent wholes, the increased centrality in the two MFG nodes could likewise reflect effortful or extensive episodic memory search during processing of high elaboration events.

Activity in a region in the left superior frontal gyrus (L-SFGi) was on the other hand related to subsequent forgetting, regardless of levels of processing. The node is however not considered part of the default memory network, which could be expected from its negative relationship with encoding, but the region is typically thought to be a part of a dorsal network which supports top-down executive control processes^58^. It can be speculated that the results reflect competition between processes that support encoding and processes required for the control of the task, such that on trials demanding more cognitive control for task switching (i.e. items that are rarely associated with lifting/eating), fewer resources were available for encoding. Similar activity pattern has been reported by Otten *et al*.^17^ and Kim^10^.

When examining the univariate fMRI analyses (Fig. 4), we find that this left frontal region shows a negative subsequent source memory effect in concert with the right frontal, and parietal/occipital regions showing positive subsequent memory effects. Still, these regions all display similar changes in communication patterns, and take more central roles in the network during deep processing. One interpretation of these seemingly contradictory findings is that although a region may have a minor, or even negative association with memory encoding when studied in isolation, it may still have great influence on the integration and control of information flow in the brain.

The tested centrality measures (see supplementary material for degree, EC, BC results) do differ in what network attributes are weighted when a node’s importance or centrality is evaluated. Degree and CC are linear measures of the number of strong connections to other nodes, and how tightly connected a node on average is to all other nodes in the network, respectively. EC does on the other hand reflect to what degree a node communicates with other important nodes, following the somewhat recursive idea that important nodes are important because they communicate with other important nodes. A node high in BC is central in a network because other nodes communicate through the node, the node is often on the shortest paths between other nodes^48^. When we found elaboration-related changes in all centrality measures but BC, this may reflect that elaboration does not simply affects the breadth of communication, for example through more numerous connections with sensory regions, but that the regions possibly increase communication with other well-connected nodes to a greater degree during high elaboration.

Why is increased centrality of the elaboration network nodes during encoding associated with enhanced source memory performance? The findings could potentially be informative of how elaboration enhances memory. Individuals may differ in their predisposition or ability to use elaboration as a memory enhancing strategy, which in turn could be reflected as increased centrality. The centrality - source memory relationship can reflect increased integration between the constituent parts of events or scenes, or enhanced strength of the memory trace itself through iterated elaborations. Age related changes in brain and cognition has been shown to affect the use and usefulness of semantic encoding strategies^59,60^, and it is possible that the individual differences in the neural connectivity patterns and memory performance we observe also reflect inter-individual differences in encoding strategies or cognitive abilities.

Schott^6^ examined connectivity associated with elaboration specifically and found that high elaboration was associated with increased connectivity between the hippocampus and bilateral ventrolateral prefrontal cortex and right temporoparietal junction. The findings of increased connectivity between hippocampus and distributed cortical regions is a hallmark of episodic memory function and is consistent with several theories of memory^61–63^. In the present study, we did not find increased centrality in hippocampus in any condition, i.e. changes in hippocampus centrality was not significantly associated with either subsequent memory, elaboration or congruity. While unexpected, several studies of anatomical and functional connectivity using graph theoretical measures have failed to assign the importance to the hippocampus in the brain network as one would expect considering the import role of the hippocampus in episodic memory function^21,64,65^, and these null-findings exemplify the point made above, that when regions are studied in the context of network interactions, the results can differ from what is found when the activity of the same regions are studied in isolation.

Differences in network edge configurations were observed for elaboration, but not congruity. If congruity affects encoding simply through increased elaboration, we would expect to see similar activation and connectivity patterns for both congruent and high elaboration events. The data does not provide convincing support for this explanation. Rather, the results indicate that while both elaboration and congruity benefit episodic memory encoding, the functional brain correlates of these processes are not identical. The results thus detail the brain activity correlates of critical processes for episodic memory encoding, well-established at the behavioral level in the LOP framework^1,2^, and demonstrate that they may have different communication patterns, and possibly independent effects on memory encoding.

## Limitations

Similar to the tasks used in other studies on levels of processing^6,17^, it can be argued that while difference in depth exists, the low elaboration condition in the present study still entails some degree of semantic elaboration, but in a different intensity than the high elaboration condition. We do however find the behavior results we would expect with regard to LOP theory.

## Conclusion

In sum, the results demonstrate that while elaboration and congruity have large but similar effects on memory for both item and context, the cortical signatures of these processes are not identical. Centrality in a distinct set of cortical regions was associated with level of elaboration, but no such association was found for congruity. Further, in the same set of regions, we found a relationship between centrality increase during episodic memory encoding and episodic memory ability, indicating that elaboration as a feature of episodic memory encoding has real importance beyond semantic classification. Future studies should test whether changes in network properties during high vs. low elaboration can be identified during development and aging.

## Supplementary information

Supplementary information

## Data Availability

The dataset analyzed during the current study is available from the corresponding author on reasonable request.
