# Supplementary material for: Elaboration Benefits Source Memory Encoding Through Centrality Change
Source: Sci Rep. 2019 Mar 6;9:3704. doi: 10.1038/s41598-019-39999-1 (PMC6403239; doi:10.1038/s41598-019-39999-1)
Supplement: Supplementary file 1 — Supplementary information [file 41598_2019_39999_MOESM1_ESM.pdf]

Elaboration Benefits Source Memory Encoding Through Centrality Change - Supplementary Information

Inge K. Amlien[1,], Markus H. Sneve[1], Didac Vidal Piñeiro[1], Kristine B. Walhovd[1,1], Anders M. Fjell[1,1]

1. Methods

1.1. Degree

The *Degree centrality* of a node is simply the number of direct connections from the node to other nodes, or the number of nodes at a distance 1. The most highly connected nodes are assumed to have strong influence over other nodes in the network, by the the high degree of connectivity and central placement in the network [48].

1.2. Betweenness Centrality (BC)

The BC of a node is defined as the fraction of shortest paths between any pair of nodes that travel through the node. BC describes to what degree other nodes needs to travel through the node, on the shortest path to other nodes in the network. A node high in BC is important because it can function as a gatekeeper for information, in other words, act as a bridge between regions. The betweenness centrality of a node v is given as:

$$BC(v) = \sum_{s \neq v \neq t} \frac{\sigma_{st}(v)}{\sigma_{st}}$$

where σ_{st} is the total number of shortest paths from node s to node t and $\sigma_{st}(v)$ is the number of those paths that pass through v.

1.3. Eigenvector Centrality (EC)

The EC of a node depends both on the number of edges, and on the quality of the connected neighboring nodes. Nodes that connect to other nodes that also have high EC, have themselves high EC. This measure is used by for example Googles PageRank algorithm, with the idea being that a node is important if its neighbours are important.

The EC of a node (v) can be calculated as:

$$EC(v_i) = \frac{1}{\lambda} \sum_{v_j \in N(v_i)} a_{v_i,v_j} \times EC(v_j)$$

where $a_{i,j}$ is the entry in the adjacency matrix A, denotes the set of neighbours of v_i, and is constant [48].

Email address: inge.amlien@psykologi.uio.no (Inge K. Amlien)

2. results

2.1. Node Degree

Figure S1 shows mean degree across all thresholds, for the high vs low elaboration contrast. A set of four nodes showed higher degree during high vs low elaboration (Figure 7). The set of nodes were three frontal nodes (L-SFGi, R-MFG-a, R-MFG-p) and one posterior node (R-IPL). Five nodes also showed the opposite effects, i.e. higher degree during low elaboration compared to high elaboration (L-HC, L-STGp, L-SFGs, L-OFC, R-MTGa)

2.2. Eigenvector Centrality

For EC, three of the four nodes associated with the degree and CC were also increased during the high vs low elaboration. The three nodes were L-SFGi, R-IPL and R-MFGp (Figure S2). L-OFC was the only node showing the opposite pattern, with increased EC during low compared to high elaboration.

2.3. Betweenness Centrality

For betweenness centrality, none of the nodes were found to display reliably increased centrality during the high vs low elaboration (Figure S3).

2.4. Centrality - Source memory performance

The centrality - source memory performance correlations for degree were r = .38, $p < .001$, and for EC r = .29, p = .002). For BC, no nodes showed centrality increase with LOP, so further analysis was run. The scatterplots and permutation distributions for degree and EC are shown in Figure S4.

2.5. Congruity and Subsequent Source memory

We repeated the analyses with the contrasts [high congruity > low congruity] and [source memory > no source memory] and found no nodes that showed significantly increased centrality (Figure S5).

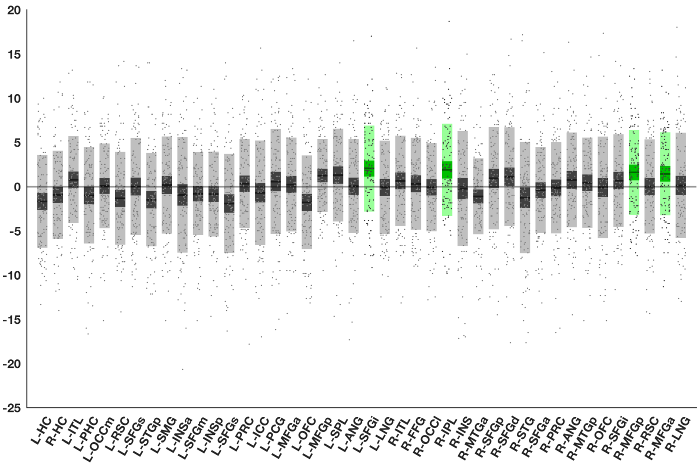

Figure S1: Nodes (X-axis) in green show significantly increased degree in the high-elaboration vs low elaboration condition. The values on the Y-axis represent mean degree change between conditions, averaged across all thresholds. Dots represent individual data points, horizontal lines represent group means, dark area 95% confidence interval, light area standard deviation.

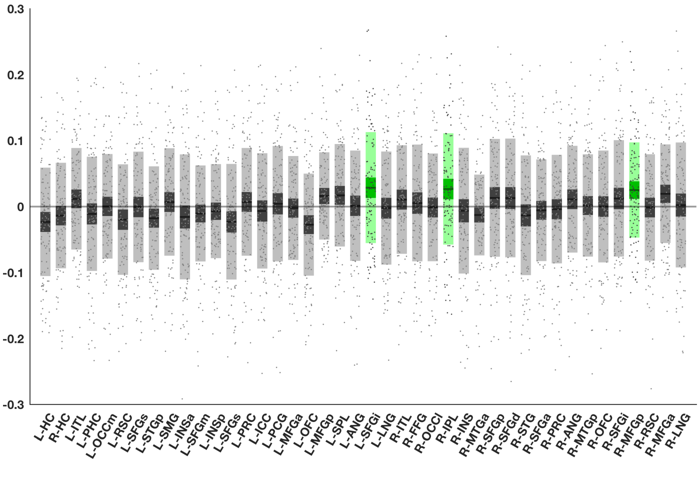

Figure S2: Nodes (X-axis) in green show significantly increased EC in the high-elaboration vs low elaboration condition. The values on the Y-axis represent mean EC change between conditions, averaged across all thresholds. Dots represent individual data points, horizontal lines represent group means, dark area 95% confidence interval, light area standard deviation.

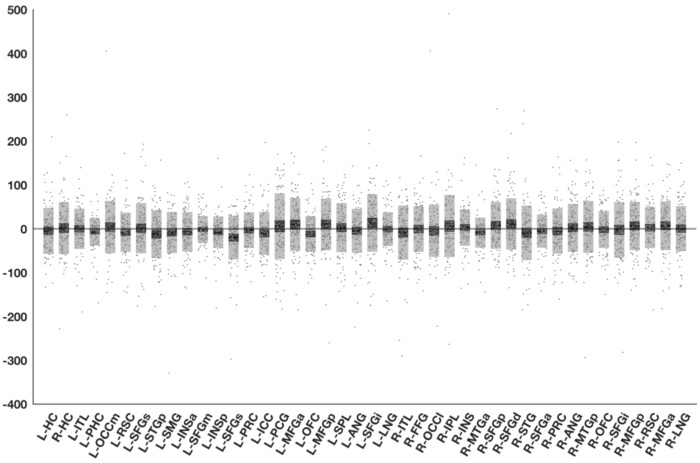

Figure S3: Nodes (X-axis) with values on the Y-axis representing mean betweenness centrality difference between high and low elaboration, averaged across all thresholds. Dots represent individual data points, horizontal lines represent group means, dark area 95% confidence interval, light area standard deviation.

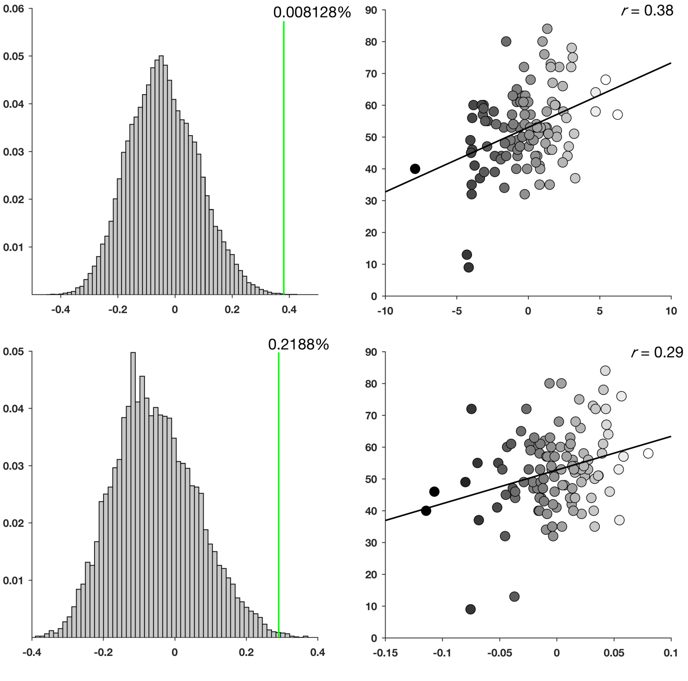

Figure S4: Left column; Null distribution of r-values (exhaustive permutations) between connectivity and source memory, using constellations of nodes not in the elaboration network. Correlation value from right side shown as green line. Right column; Mean connectivity difference between subsequent source, and no subsequent source memory across elaboration network nodes of interest represented on the X-axis. Corrected source memory performance on the Y-axis. top = degree, bottom = EC.

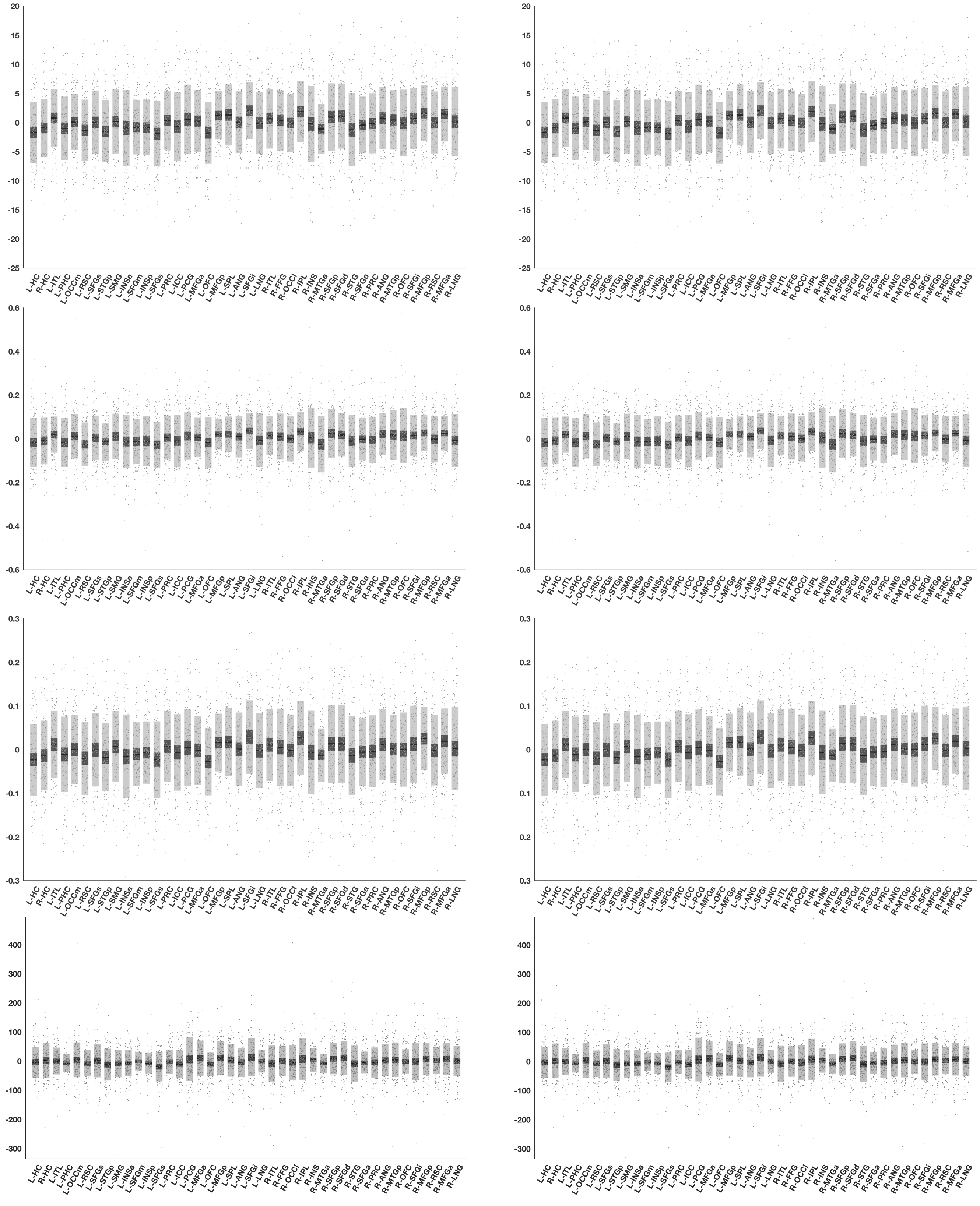

Figure S5: Left column; High congruity – low congruity. Right column; Source memory – no source memory. Top to bottom; Degree, CC, EC, BC. The values on the Y-axis represent centrality change between conditions, averaged across all thresholds. Dots represent individual data points, horizontal lines represent group means, dark area 95% confidence interval, light area standard deviation.
